# Anesthetic Approaches and Perioperative Complications of Total Hip Arthroplasty in Gaucher Disease: A Control-Matched Retrospective-Cohort Study

**DOI:** 10.3390/life13081716

**Published:** 2023-08-10

**Authors:** Ariel Grass, Eyal Riemer, Ari Zimran, Shoshana Revel-Vilk, Andres Freundlich, Ehud Lebel, Alexander Ioscovich

**Affiliations:** 1 Department of Anesthesia, Shaare Zedek Medical Center, 12 Shmuel Bait St., P.O. Box 3235, Jerusalem 9103102, Israel; andresf@szmc.org.il (A.F.); aioscovich@szmc.org.il (A.I.); 2 Faculty of Medicine, The Hebrew University of Jerusalem, Ein Kerem. P.O. Box 12271, Jerusalem 9112102, Israellebel@szmc.org.il (E.L.); 3 Gaucher Disease Unit, Shaare Zedek Medical Center, 12 Shmuel Bait St., P.O. Box 3235, Jerusalem 9103102, Israel; 4 Department of Orthopedic-Surgery, Shaare Zedek Medical Center, 12 Shmuel Bait St., P.O. Box 3235, Jerusalem 9103102, Israel

**Keywords:** intraoperative complications, Gaucher disease, general anesthesia, total hip arthroplasty, regional anesthesia

## Abstract

Objectives: Gaucher disease’s (GD) pathophysiology generates anesthetic concerns in total hip joint arthroplasty (THA), and due to its rareness, data on perioperative risks are scarce. This 22-year study at a large reference center addresses anesthetic management and perioperative outcomes in GD. Methods: This retrospective-cohort study assessed anesthetic success and safety in 30 THA patients, comparing them with a control-matched group. Data on clinical characteristics, perioperative events, and outcomes were collected. The primary outcome was the success rate of anesthesia induction performance at first attempt. Secondary outcomes were difficult intraoperative course and hemodynamic management, and the development of postoperative complications. The age, sex, weight, body mass index, and primary-to-revision hip arthroplasty ratio were similar in both groups. Results: There was no significant difference at all-type anesthesia first initiation attempt success. No particular preference by staff anesthetists for general anesthesia or neuraxial procedures was observed. The GD group showed a significantly higher mean of intraoperative packed Red Blood Cell units administered ((0.73 vs. 0.18); (*p* = 0.038)), higher intraoperative and postoperative platelet transfusion incidence ((5/30 [16.7%] vs. 0/56 [0.00%]; *p* = 0.004) and (3/30 [10%] vs. 0/56 [0%]; *p* = 0.040)), and longer mean recovery room length of stay (426 ± 412 vs. 175 ± 140; *p* = 0.004). Postoperative complications were not significantly different.

## 1. Introduction

Gaucher disease (GD) is probably the most common lysosomal storage disease [1] and can greatly affect a patient’s health-related quality of life. The most severe symptoms include bone pain and persistent tiredness, which can disrupt one’s ability to attend school, work, and social events. Its etiology is an autosomal recessive mutation causing a defect in the lysosomal enzyme β-glucosidase, leading to the accumulation of the non-degraded glucocerebroside in macrophages, mainly in the reticuloendothelial system (spleen, liver, and bone marrow)**.** There are three clinical presentation groups: Type I is an adult chronic form that is non-neuropathic and the most common. Type II is an acute neuronopathic disease that leads to early mortality. Type III is a juvenile sub-acute neuronopathic form [2]. This differentiation is important as symptomatic expressions are more common in severe neuronopathic forms of the disease. Bone involvement can occur in any bone, particularly of the proximal and distal femur, proximal tibia, and proximal humerus, and manifest in the form of pathological fractures, vertebral compression fractures, and osteolytic lesions [3,4]. Osteonecrosis leading to osteoarthritis of large joints is relatively common in GD, with an increased incidence of hip joint osteoarthritis [5]. While the introduction of enzyme replacement therapy (ERT) has led to a substantial reduction in osteonecrosis development [6], adult patients previously diagnosed with Gaucher disease before ERT was available could still develop it while on therapy. Total hip arthroplasty (THA) is occasionally offered for symptomatic patients at younger ages [7].

Anesthetic management concerns are raised during the pre-anesthesia planning phase due to theoretically higher risks from vital structures deposition. Airway management concerns for anesthesia can have an implication for securing the patient’s airway and oxygenation and are based on reported respiratory dysfunction, hypoxemia, and altered pulmonary function tests [8,9,10,11,12,13], with possible bodily posture affection due to both pre-existing neurological symptoms and potential adverse reactions to dopaminergic agents [14]. Hemodynamic stability during anesthesia can be impacted in the presence of cardiac affections, and the presence of valvulopathies and arterial calcification have been reported [15,16] and could be fatally progressive. Hematological aspects of GD may affect the hemodynamic stability and selection of the anesthesia induction techniques or usage of special monitoring methods. Among these, we find a higher infection risk, anemia, thrombocytopenia, abnormal platelet aggregation, and coagulation factor deficiency [17,18,19]. All are leading concerns for surgical site infection and disproportionate or massive bleeding. These conditions may present difficulties in the consideration of both general (GA) and neuraxial anesthesia (NA), their perceived likelihood of success, and the risk of complications.

Although small sized samples, such as retrospective case series and case reports supporting the former anesthesia concerns, do exist, describing the high prevalence of Pulmonary hypertension, lack of significant airway management difficulties, a high requirement rate of blood product transfusion, higher rates of postoperative wound infections, and a significantly higher abnormal platelet aggregation preoperative prevalence [17,20], comparative studies have yet not been conducted heretofore. We hypothesized that the anesthetic management of patients with Gaucher disease undergoing hip arthroplasty would be more challenging than for non-GD patients. The Gaucher Disease Unit is the world’s largest referral center for GD, following more than 900 patients over 3 decades, and it has allowed us to conduct a control-matched retrospective-cohort study with the purpose to analyze whether difficult anesthesia initiation, intraoperative course, hemodynamic management, and postoperative complications are associated to Gaucher disease diagnosis.

## 2. Methods

### 2.1. Study Design and Aims

This is a retrospective-cohort study conducted at a tertiary-care academic health center and was approved by the local Research Ethics Board (No. 0007-19-SZMC). The aim was to evaluate the association between diagnosed Gaucher disease and difficult anesthetic management, and also the anesthetic and surgical clinical outcomes to guide the professional to tailor his preoperative anesthetic plan safely and avoid potentially foreseeable complications.

### 2.2. Study Population

This study included all patients with GD, who underwent Total Hip Replacement at a single medical center between 1 March 1997 and 1 March 2019 to comply with our power analysis requirements. The inclusion criteria were the following: (1) elective total hip arthroplasty (primary or revision); (2) a confirmed diagnosis of GD [1]; and (3) age 18 years or older. A concurrent control-group (CG) was created from SZMC’s medical records by manual-selection control-matching, which was especially performed for each GD patient. A control was selected when all of the following criteria were met: same gender, same type of surgery (primary or revision), age difference of no more than 10 years, and who had the closest surgery date to the case.

### 2.3. Sample Size and Statistical Analysis

The sample size was based on the literature’s reported incidence of difficult anesthetic intraoperative management [21,22,23]. Assuming an incidence of the primary outcome in the CG of approximately 58%, and expecting a 50% increase in the exposure group to 87%, it was estimated that 26 patients in the Gaucher disease group and 56 in the CG would be required for statistical significance with a Type I error of less than 0.05, and a power of 0.8, with an intended matching ratio of 1:2 (two control patients per exposed patient). Based on the local historical yearly procedures for GD patients, we expected to find approximately 1–2 THA surgeries for GD patients per year, obtaining approval for data collection for our retrospective review. Two-tailed *p*-values were used, considering significance as the *p*-value < 0.05. Descriptive variables were reported as frequency (%), and the mean ± SD. Qualitative variables were analyzed using the chi-square test or Fisher’s exact test. The comparison of the quantitative variables between the two groups was done by the parametric *t*-test for normal distribution data or Mann–Whitney for non-normal distribution data.

### 2.4. Data Collection

Data were extracted from clinical files, including demographic, GD-related data, comorbidities, intraoperative data, such as the type of surgery and anesthesia, vital signs, perioperative blood products consumption, duration of surgery and anesthesia, and postoperative outcomes, such as the incidence of infections, length of stay in the recovery room and in the hospital, transfer to the intensive care units, and mortality.

### 2.5. Outcome Measures

The primary outcome was the requirement rate of more than one attempt at any-type anesthesia induction technique, including general anesthesia with endotracheal intubation or any-type of neuraxial anesthesia procedure. The secondary outcomes included: (1) number of attempts of endotracheal intubation; (2) number of attempts of neuraxial anesthesia; (3) volume of intraoperative crystalloids and colloids infused; (4) mean intraoperative change in hemoglobin and platelet levels; (5) use of invasive monitoring, such as intra-arterial blood pressure or central venous pressure measurements; (6) duration of surgery and anesthesia; (7) perioperative blood products transfusion incidence and volume; (8) prolonged length of stay in the recovery room; (9) intensive care unit (ICU) transfer; (10) prolonged length of stay in the hospital; (11) postoperative prolonged antibiotic treatment beyond a single prophylactic dose; (12) thromboembolic events incidence; and (13) 30-day in-hospital all-cause mortality rate.

## 3. Results

A total of 30 patients with Gaucher disease and 56 matched controls were identified meeting the inclusion criteria over a 22-year period. Although Total Hip Replacement is uncommon in young non-GD patients (CG), the matching quality was deemed satisfactory, with a final matching ratio of 1:1.87 attained, maintaining an age-similarity matching criterion of a maximum of 15 years instead of increasing the maximum allowed age difference with the CG.

### 3.1. Patient and Clinical Characteristics

The age, sex, weight, body mass index, primary-to-revision THA, type of anesthesia, preoperative hemoglobin level, and platelet counts were similar in both groups (Table 1). The most frequent GD mutations were N370S/N370S (the most common and milder genotype among patients with Type I GD presented in 16/28 [57.1%]), while none had a neuronopathic (Type III) diagnosis.

### 3.2. Anesthesia Induction

For the primary outcome, a requirement rate of more than one attempt at any-type anesthesia techniques was not significantly different between both groups, having occurred in one patient with GD (3.3%) and 9 controls (16.1%); (*p* = 0.155), and neither was the total number of any-type anesthesia attempts (1.03 vs. 1.16, respectively). The independent incidences of requiring more than one attempt at endotracheal intubation or neuraxial anesthesia were not different either ((1.0 vs. 1.0); (*p* = 1.000) and (5.6% vs. 12.2%); (*p* = 0.656), respectively). Both the decision to use invasive monitoring techniques and the selection of neuraxial over general anesthesia were not significantly different in the Gaucher disease group for the study period (Table 2).

### 3.3. Surgical and Hemodynamic Management

While both the duration of anesthesia and the duration of surgery were longer in the GD group, they were not significantly different. Platelet transfusion occurred intraoperatively in significantly more cases in the GD group, as it was present in five patients (16.7%) but none of the CG (*p* = 0.004). Accordingly, the mean intraoperative dose of platelet units given (U) was higher as well ((0.38 vs. 0.0); *p* = 0.002). There were no differences in the mean postoperative-preoperative hemoglobin difference, platelet levels, or the amounts of crystalloids or colloids given. Although the intraoperative transfusion incidence of packed Red Blood Cells (pRBC) was higher in patients with GD (26.7%) than in CG patients (10.7%), this was not significant. Nevertheless, there was a significantly higher mean amount of packed Red Blood Cells units given intraoperatively to the GD group vs. the CG ((0.37 vs. 0.18); (*p* = 0.038)). Intraoperative change in hemoglobin and platelet levels were not significantly different. Additional intraoperative data can be seen in Table 3.

### 3.4. Postoperative Complications

The mean recovery room length of stay was significantly longer in the Gaucher group (426 vs. 175 min; *p* = 0.004), but the mean in-hospital length of stay and the incidence of transfer to the Intensive Care Unit (%) were similar. The incidence of postoperative platelets administration was significantly higher in the Gaucher disease group (3/30 [10%] vs. 0/56 [0%]; *p* = 0.040), with a mean amount of platelets transfused significantly higher as well (0.87 ± 4.38 (0–24) vs. 0 ± 0.00 (0-0); *p* = 0.017). The incidence of postoperative requirements of packed Red Blood Cells was lower in the GD group, but not statistically significant, while the mean number of units given were similar in both groups. For the studied postoperative complications, the thromboembolic events incidence, postoperative massive transfusion incidence, and mortality incidence were non-significantly different between the GD group and the CG. Similarly, the incidence of prolonged antibiotic treatment beyond the first prophylactic dose was not significantly different. Additional statistical data are presented in Table 4.

## 4. Discussion

To our knowledge, this study is the first large control-matched retrospective-cohort study to address anesthetic induction techniques, intraoperative management, and complications in the GD population undergoing THA. Due to the scarcity of data and rarity of the disease limiting the prevalence of cases to be reported on, even with our center being a GD reference center, and to meet our power analysis, the study was conducted during a long observation period of 22 years, privileging increasing the case number than reducing the total number of years of the study. When increasing the study period, the risk of bias could theoretically increase from changes in medical treatment, and this needs to be considered by the reader while interpreting these results; however, it is the author’s opinion that general anesthesia and neuraxial anesthesia induction techniques have not significantly changed during this study period.

Due to the lack of data in the medical literature in the GD population, additional to the primary outcome, multiple secondary outcomes were included for exploratory purposes to make the available data accessible and to orient future studies.

### 4.1. Anesthetic Technique

The current study evaluated the challenges of anesthesia for patients with Gaucher disease, as compared to those not affected by Gaucher disease, while undergoing hip joint arthroplasty. This study’s findings reinforce the previous literature reports [24], as the study found no significant difference in the composite primary outcome, in the difficulty to succeed at a first initiation attempt of anesthesia by either endotracheal intubation or neuraxial anesthesia versus the control group, or in each technique’s independent analysis. Gaucher disease patients who underwent the procedure under general anesthesia did not present greater difficulty in ventilating and establishing a secure airway with an endotracheal tube than the control patients, and no patient required more than one intubation attempt. Although the theoretical concern of anesthetic induction and maintenance conditions were affected by Glucosylceramide deposits and the presence of neurological signs, no particular preference by staff anesthetists for GA or any neuraxial technique was found for the study period.

First, these results contrast airway concerns based on the priorly reported presence of neurological symptoms in these patients, such as trismus and opisthotonic posturing, limb stiffness, bulbar signs, convulsions [14], the theoretical concerns of upper airway glycolipid pharyngolaryngeal infiltration, and of lower safe-apnea time due to reported lung pathology in the form of interstitial lung disease and hepatopulmonary syndrome [9,10], and ventilatory reserve affection from reductions in functional residual capacity, total lung capacity, and alveolar-capillary diffusion abnormality [8]. Second, the authors acknowledge the potential of bias in this airway management finding due to the documentation method used at this center, since the anesthetic chart requests marking the field “Difficulty at endotracheal intubation?”. If present, marking omissions could possibly mean either a negative response or rather the lack of documentation of this finding. It was chosen to assume a lack of marking as a lack of difficulty, i.e., as success in a single attempt, considering that in our center, the documentation of difficult intubation is commonly registered when present. Additional difficult intubations that were not reported cannot be ruled out, which would apply to both study groups. In patients in whom neuraxial anesthesia was performed, no significant difference could be seen in the number of attempts. In the Gaucher group, there was only one patient (0.03%) who required more than one attempt, compared to five patients (0.09%) in the CG. Those findings suggest that airway and spine conditions might not significantly impact anesthetic success, which is supported by the lack of association to perioperative anesthetic complications. This could be interpreted as a lack of significant Glucosylceramide deposition in glottic/supraglottic soft tissues and those superficial to the lumbar spine, if existent. A second possibility could be meticulous anesthesiologist preparation. The same is true for neuraxial anesthesia (NXA) as an expected difficulty in performing neuraxial anesthesia due to osteopathological changes, and the suspicion of an increased risk of bleeding and postprocedural epidural hematoma development in the context of a suspected coagulation abnormality could be worrisome and deterring to anesthetists. Additionally, the potential affection of accessory muscles that neuraxial anesthesia can occasionally cause when reaching higher spinal nerves blockage under a possible reduced respiratory reserve could cause apprehension. These factors, however, did not seem to affect the clinical decision to perform NXA, nor the technical difficulty or complications, implying that both techniques might be equally successful in GD patients.

In both cases, this suggests that a higher index of suspicion for anesthesia complications may not be particularly required. No contraindication to either general or neuraxial anesthesia, nor the usage of any specific drug, has been suggested by the current literature, and our study’s results do not suggest the contrary. Rather, the decision regarding which anesthetic techniques or drugs should be used must solely be impacted by usual specific contraindications, such as the presence or absence of coagulopathy or concurrent anticoagulation for neuraxial procedures.

One should consider these findings cautiously, specifically the retrospective nature of the study. Prospective studies may substantiate these findings, although this might be difficult while considering the paucity of this illness. As for the secondary outcomes, the duration of anesthesia was not significantly longer among Gaucher patients. Although this study did not analyze specific intraoperative oxygenation or mechanical ventilation parameters, it did not find an association between Gaucher disease and postoperative pulmonary complications, nor other significant complications.

### 4.2. Blood Products

Due to a lack of GD-specific intraoperative blood transfusion guidelines, the decision to administer packed Red Blood Cells was taken by treating anesthesiologists according to the same criteria used for any patient without GD based on his professional criteria. The packed Red Blood Cell intraoperative transfused amount was significantly higher in the GD group, but was not significantly lower postoperatively. Intraoperatively, approximately one in four GD patients received packed Red Blood Cells, in contrast to approximately one in ten in the CG (*p*-value: 0.07). This difference could be explained by the disease’s own risk factors for bleeding, however, we cannot rule out a more liberal approach while treating these patients as an explanation, affecting the anesthetist’s decision to administer blood products. Postoperatively, this was inverted, with approximately one in four Gaucher patients receiving packed Red Blood Cells during their recovery room stay, compared with approximately one in two control patients. An average of 0.57 ± 1.22 units were given postoperatively per Gaucher disease patient, compared with an average of 0.88 ± 1.48 per control patient (*p* = 0.133). Among the possible reasons for this contrasting finding, the simpler explanation might be the better hemodynamic optimization of GD patients from higher intraoperative administration, therefore leaving them more properly compensated postoperatively than the control group. Unfortunately, blood loss data collection for the study period was challenging due to imprecise blood loss documentation narrowing further contextualization. Another alternative explanation is a more defensive approach by the anesthesia staff, from bleeding concerns related to the disease, thus lowering the threshold for packed Red Blood Cell administration in GD patients compared with a more conservative approach with control patients. That is, pRBCs would be administered to GD patients for milder bleeding, as opposed to the control patients, in which a more conservative approach would have been practiced, which would involve waiting for more extensive bleeding prior to deciding on the administration. Such a theory could explain the reversal of this trend, with control patients needing more blood products postoperatively than GD patients. In addition, it can be seen that there was one patient in the study group and two patients in the control group who were subjected to a massive blood transfusion protocol, defined as the requirement of five or more packed Red Blood Cell units not meeting statistical significance. To date, no work has been described in the literature reviewing specific intraoperative massive blood transfusion criteria for Gaucher disease patients. Platelet doses were given exclusively to Gaucher patients; thus, this finding was statistically significant for the study group. These results are consistent with the reported hematological manifestations of GD: lower levels of coagulation factors, hypersplenism with consequent thrombocytopenia, and impaired platelet function as potential contributors to increased surgical blood loss. It should be noted that most of these patients had a preliminary platelet administration protocol designated by a hematologist following their GD management, which was delivered in writing to the anesthesia staff from the Gaucher clinic ahead of time.

### 4.3. Extended Antibiotic Coverage

All patients received prophylactic antibiotic therapy, as is conventional for these surgeries [25]. Patients for whom antibiotic therapy was prolonged more than the conventional first prophylactic dose, or alternatively, for whom antibiotic therapy was reinitiated later in their hospitalization, were flagged as having received prolonged antibiotic therapy. Gaucher patients had almost double the therapy period than the CG, almost reaching significance (*p*-value: 0.063). Allegedly, two possible explanations could be raised for this finding: first, a more cautious approach in light of the suspected increased infection risk (especially in splenectomized patients), and second, the postoperative diagnosis of an infectious process (such as pneumonia). The patient’s chart data support that the prolongation of antibiotic treatment was mostly associated with the presence of signs of infection (e.g., fever and surgical wound findings), making the second explanation more plausible.

### 4.4. Other Findings

The most common genotype observed was N370S/N370S, which is consistent with the literature. While typically clinically milder, due to its high frequency, most patients presenting severe manifestations do belong to this group, being underrepresented. In this study, none of the patients in the GD group that presented osteonecrosis and underwent THA had neuronopathic Type III Gaucher disease. It is worth noting that the profile of patients presenting osteonecrosis before and after the Enzime Replacement Therapy initiation era could differ in the future. The recovery room length of stay was significantly longer in GD patients. (mean. Diff: 251 min). These patients may have required prolonged care for a justified clinical reason, or a cautious approach was likely taken with GD patients by recovery room staff for longer monitoring time from concerns for postoperative complications, even in the absence of a particular clinical condition requiring a specific treatment. There may be room for further research into the causes of this increased delay in the GD group.

## 5. Limitations of This Study

The study limitations are its retrospective nature, the impossibility of concluding causation, and the low number of patients that could be included in the Gaucher disease group, given that it is an infrequent disease, as well as in the CG with the selected matching criteria. Given that age was matched, we are aware that an early age CG might not be representative of the more common daily practice older Total Hip Replacement cohort due to osteoarthritis alone, with the selected younger comparator group being potentially affected by additional unknown confounders. These could lead to more or less differences than if compared with older populations, and needs to be considered. Further studies are required to confirm these results. While prospective studies are required, Gaucher disease’s rarity would significantly limit the possibility of conducting them.

## 6. Conclusions

This observational control-matched cohort-study did not demonstrate an association with the rate of difficult anesthesia induction nor a significantly higher risk of complications, suggesting that GD patients can safely undergo either GA or neuraxial anesthesia for hip joint arthroplasty. There was an association to the Gaucher disease group of higher intraoperative packed Red Blood Cell administration, an increased incidence of platelet transfusion both intraoperatively and postoperatively, and also a longer mean recovery room length of stay. The ideal preoperative, intraoperative, and postoperative transfusion criteria remain to be defined in this population. It might be prudent for anesthesiologists, whenever possible, to assess a patient’s coagulation status and blood coagulation tests preoperatively. Preoperative parameters can be improved through optimal ERT therapy and specialist management, and referring GD patients to a specialized center can provide significant value in optimizing them before anesthesia and surgical procedures.

## Figures and Tables

**Table 1:** Characteristics of the research groups.

	Gaucher (n = 30)	Control (n = 56)	*p*-Value
Age (№ ± SD [range])	48.2 ± 12.96 (17–67)	49.93 ± 12.32 (24–68)	0.544
Women/Men (№ [%])	13 [43%]/17 [57%]	26 [46%] (30 [54%])	0.365
Weight (№ ± SD [range])	73.38 ± 11.26 (41–90)	77.89 ± 16.9 (49–120)	0.230
Body Mass Index (№ ± SD [range])	27.04 ± 2.52 (22.40–31.23)	31.61 ± 6.78 (23.56–42.25)	0.085
History of osteonecrosis	25 of 30 [83.3%]	10 of 55 [18.2%]	0.001
Primary/Revision	20 [67%]/10 [33%]	41 [73%]/(15 [27%])	0.524
Preoperative Hemoglobin levels (№ ± SD [range])	13.03 ± 1.98 (6.9–16.2)	13.23 ± 1.84 (7.7–18)	0.661
Preoperative Platelet counts (№ ± SD [range])	219,750 ± 134,283.96 (21,000–679,000)	262,060.71 ± 117,820.58 (149,000–1,011,000)	0.144

Values presented. “Mean ± standard-deviation (Range)”, or as the number of cases [Percentage]. Frequency presented as numbers while percentages are calculated from available data. *p*-values are calculated using *t*-test. *p* < 0.05 was deemed statistically significant.

**Table 2:** Types of anesthesia and invasive monitoring used.

	All Patients (n = 86)	Gaucher (n = 30)	Control (n = 56)	*p*-Value
Arterial line used. № (%)	5 (5.8%)	2 (6.7%)	3 (5.4%)	1.00
Central venous line access used. № (%)	2 (2.3%)	2 (6.7%)	0 (0.0%)	0.119
Performance of any neuraxial anesthesia technique. № (%)	58 (68%)	18 (60%)	40 (71.4%)	0.576

Values presented. “Mean ± standard-deviation (Range)”, or as the number of cases [Percentage]. Frequency presented as numbers while percentages are calculated from available data. *p*-values are calculated using Fisher’s exact test. *p* < 0.05 was deemed statistically significant.

**Table 3:** Intraoperative data: duration, crystalloids blood products usage, and laboratory results.

	Gaucher (n = 30)	Control (n = 56)	*p*-Value
Duration of anesthesia (mins ± SD)	180 ± 80	160 ± 55	0.230
Duration of surgery (mins ± SD)	141 ± 76	120 ± 49	0.171
Mean crystalloids given (mL ± SD [range])	2350 ± 1328.92 [0, 5000]	2261.11 ± 966.5 [600, 4500]	0.749
Mean colloids given (mL ± SD [range])	444.44 ± 481.69 [0, 1500]	700 ± 388.06 [500, 1800]	0.089
Intraoperative massive transfusion incidence (%)	3.3% (1/30)	0% (0/56)	0.349
Intraoperative pRBC transfusion incidence (%)	26.7% (8/30)	10.7% (6/56)	0.070
Intraoperative mean pRBC units given (№ ± SD [range])	0.37 ± 1.39 [0, 5]	0.18 ± 0.54 [0, 2]	0.038
Mean hemoglobin change, preoperative-postoperative (g/dL ± SD [range])	−2.49 ± 1.69 [−4.9, 2.4]	−2.58 ± 1.50 [−6.5, 1.2]	0.794
Intraoperative platelets transfusion incidence (%)	16.7% (5/30)	0.0% (0/56)	0.004
Intraoperative mean platelet units given (U ± SD [range])	0.38 ± 2.9 (0, 12)	0.0 ± 0.0 (0, 0)	0.002
Mean platelet change preoperative-postoperative (plt/μL ± SD [range])	−29,773.37 ± 73,366.01 [−216,000, 169,000]	−24,150 ± 68,826.07 [−245,000, 202,000]	0.734

Values presented. “Mean ± standard-deviation (Range)”, or as the number of cases [Percentage]. Frequency presented as numbers while percentages are calculated from available data. *p*-values are calculated using the chi-square test. *p* < 0.05 was deemed statistically significant.

**Table 4:** Postoperative outcomes and complications.

	Gaucher (n = 30)	Control (n = 56)	*p*-Value
Mean recovery room length of stay (mins ± SD [range])	426 ± 412 [70, 1185]	175 ± 140 [0–1020]	0.004
Mean hospital length of stay (days ± SD [range])	9.97 ± 3.61 [4, 19]	11.75 ± 11.76 [4, 71]	0.449
PACU morphine dose given (mg ± SD [range])	11.79 ± 3.82 [8–20]	11.68 ± 4.68 [4, 20]	0.929
Incidence of transfer to ICU (%)	6.7% (2/30)	1.8% (1/56)	0.278
Incidence of a thromboembolic event (%)	6.7% (2/30)	0.0% (0/56)	0.119
Mortality (%)	0% (0/30)	0% (0/56)	1.000
Postoperative massive transfusion incidence (%)	3.3% (1/30)	3.6% (2/56)	1.000
Postoperative pRBC administration incidence (%)	23.3% (7/30)	42.9% (24/56)	0.072
Postoperatively mean pRBC units given (№ ± SD [range])	0.57 ± 1.22 [0, 5]	0.88 ± 1.48 [0, 8]	0.133
Postoperative platelets administration incidence (%)	10% (3/30)	0% (0/56)	0.040
Postoperative mean platelets units given (№)	0.87	0.00	0.017
Incidence of prolonged antibiotic treatment beyond a single prophylactic dose (%)	46.7% (14/30)	26.8% (15/56)	0.063

Values presented. “Mean ± standard-deviation (Range)”, or as the number of cases [Percentage]. Frequency presented as numbers while percentages are calculated from available data. *p*-values are calculated using Fisher’s exact test or chi-square test. *p* < 0.05 was deemed statistically significant.

## Data Availability

The data presented in this study are available on request from the corresponding author. The data are not publicly available due to privacy.
